# Metabolic insights into the pathophysiology of tuberculous meningitis in FFPE postmortem human brain tissue

**DOI:** 10.1007/s11306-026-02511-8

**Published:** 2026-08-07

**Authors:** Abisola R. Isaiah, Du Toit Loots, Aurelia A. Williams, Stefan Dan Zaharie, A. Marceline Tutu van Furth, Martijn van der Kuip, Shayne Mason

**Affiliations:** 1 Biomedical and Molecular Metabolism Research, Faculty of Natural and Agricultural Sciences, North-West University, Potchefstroom, South Africa; 2 Department of Anatomical Pathology, Faculty of Medicine and Health Sciences, Stellenbosch University, Cape Town, South Africa; 3 National Health Laboratory Services, Tygerberg Hospital, Cape Town, South Africa; 4 Pediatric Infectious Diseases and Immunology, Amsterdam University Medical Center, Academic Medical Center, Emma Children’s Hospital, Amsterdam, the Netherlands

**Keywords:** Tuberculous meningitis (TBM), Central nervous system (CNS) disease, Postmortem, Brain tissue, Granuloma, *Mycobacterium tuberculosis* (*Mtb*), Formalin-fixed paraffin-embedded (FFPE), Metabolic, Metabolomics, Metabolite, GC×GC-TOFMS

## Abstract

**Background:**

Tuberculous meningitis (TBM), caused by Mycobacterium tuberculosis, is the most severe form of extrapulmonary tuberculosis and isassociated with high morbidity and mortality, particularly when diagnosis is delayed. Improved understanding of the metabolic alterations associated withTBM may support the development of novel diagnostic biomarkers and provide insights into disease pathophysiology.

**Method:**

In this study, we applied anuntargeted two-dimensional gas chromatography–time-of-flight mass spectrometry (GC×GC-TOFMS) metabolomics approach to formalin-fixed, paraffin-embedded (FFPE) postmortem human brain tissue from 41 TBM cases and 36 tissue sections from 6 non-TBM control cases. Metabolomics data wereprocessed, normalized, and analyzed using multivariate and univariate statistical approaches, including principal component analysis (PCA) and partialleast squares–discriminant analysis (PLS-DA), with variable importance in projection (VIP) scores. These results were further correlated with patient clinical data.

**Results:**

Distinct metabolic profiles were observed between TBM and control tissues. Several metabolites were significantly reduced in TBM samples, particularly within the alkane and alkene classes, with additional decreases observed in metabolites associated with alcohols, fatty acids, lipids, carbohydrates, and amino acids. These metabolic alterations suggest substantial perturbations, primarily in the host lysine degradation pathway (linked to the kynurenine pathway), in TBM-affected brain tissue.

**Conclusion:**

Collectively, these findings provide insight into the metabolic landscape of terminalTBM and suggest potential metabolic pathways that may contribute to disease pathophysiology. Further investigation of these metabolic signatures in accessible patient tissue and biofluids may support the development of biomarkers and inform future therapeutic strategies for TBM.

**Supplementary Information:**

The online version contains supplementary material available at https://doi.org/10.1007/s11306-026-02511-8.

## Background

Tuberculous meningitis (TBM) is the most severe extrapulmonary form of tuberculosis (TB) and is a central nervous system (CNS) disease caused by *Mycobacterium tuberculosis* (*Mtb*) (Organization, 2024). Accurately diagnosing TBM remains a major challenge because it presents with clinical symptoms similar to other meningeal diseases, and the pathogen’s microbial load in the cerebrospinal fluid (CSF), which is considered the gold standard sample matrix for diagnosis, is usually very low and requires highly sensitive detection tools (Donovan et al., 2026). Furthermore, many patients still suffer permanent neurological disabilities or die, despite the best treatment efforts. The severity of the disease, inadequate diagnostics, and poor treatment outcomes warrant further exploratory research using alternative research strategies, such as “omics” technologies, across a variety of available sample matrices, to understand the disease’s pathophysiology and pathogenesis. A breakthrough in this area could lead to significant clinical improvements, as early detection of TBM in patients with early onsets would allow treatment to commence early enough to improve clinical outcomes.

The patient sample matrices previously analyzed in modern analytical research include urine, CSF, plasma, serum, and brain tissue (Isaiah et al., 2024; Kumar et al., 2012). The latter poses a major limitation, since the acquisition of human brain tissue is only possible after the patient’s demise, and because of the cost of storage (if employing cold storage), cumbersome ethical clearance requirements, and challenges in post-acquisition analyses with formalin-fixed, paraffin-embedded (FFPE) tissue. Limited omics research has been conducted on FFPE tissue, and only recently has a standardized protocol been published for such (Isaiah et al., 2024). Despite the advancements made in research over time, there are to date still no specific validated biomarkers which can be applied towards the early detection or diagnosis of TBM in adults (although there are some in early diagnosis of the disease in children) (Manyelo et al., 2022). There is also a paucity of understanding of the disease pathophysiology (Davis et al., 2019, Jain et al., 2018), and the formation of organized immune clusters known as granulomas in the brainstem and subarachnoid space, which primarily distinguishes TBM from other forms of meningitis histologically (Davis et al., 2019, El-Kebir et al., 2013), has recently sparked curiosity about granuloma composition and formation (Sholeye et al., 2022).

Untargeted metabolomics is a useful tool for generating hypotheses and has been applied across a range of fields to elucidate disease mechanisms. When conducting an untargeted metabolomics study, it is important to consider the instrument’s sensitivity and specificity. Existing metabolomics studies have identified metabolites associated with TBM infection, such as myo-inositol, leucine, and lysine; however, these metabolites have yet to be validated (Isaiah et al., 2025). Commonly used equipment for untargeted metabolomics studies includes two-dimensional gas chromatography coupled with time-of-flight mass spectrometry (GCxGC-TOFMS) and proton nuclear magnetic resonance spectroscopy (^1^H-NMR). The former is more sensitive and better suited for analyzing small sample quantities. In this study, we performed an untargeted metabolomics analysis using GCxGC-TOFMS on post-mortem FFPE human brain tissue to metabolically characterize TBM granulomatous brain tissue relative to non-TBM controls.

## Materials and methods

### Sample collection, criteria for selection, and ethical considerations

A cohort of FFPE human brain tissue samples was obtained from the archives of the neuropathological unit at Tygerberg Hospital, Cape Town, South Africa, and collected between 1975 and 2012. A total of 77 samples were collected, comprising 41 samples from individual TBM cases and 36 blocks of non-TBM control samples from six control patients (i.e., at least 3 tissue samples were taken from each control patient’s FFPE brain, yielding 36 total control samples) (Table 1). The TBM samples were from patients who died from TBM and had well-formed granulomas in the brain. Blocks without well-formed granulomas were excluded from this study. The control samples were taken from patients negative for any brain infection who died in accidents and whose samples underwent routine autopsy. Underlying conditions in three of the controls were congenital abnormalities, fetal alcohol syndrome, and hypoxic-ischemic brain injury (making up 11 blocks of the control samples), while the other 3 were normal brain tissue with 15 blocks. Therefore, the control samples were classified as non-TBM rather than completely healthy brains.

At autopsy, whole brains were immersed in buffered formalin. After fixation, the brains were sectioned, and between one and sixteen tissue samples per patient were collected from macroscopic regions of interest. The tissue blocks were then subjected to additional formalin fixation and paraffin embedding, after which they were stored at room temperature. Ethical clearance was obtained from the North-West University’s Ethics Committee (approval number NWU-00482-20-A1) and from the Health Research Ethics Committee of the Faculty of Medicine and Health Sciences at Stellenbosch University, Cape Town, South Africa (ethics approval numbers S12/11/298 and N09/07/185).

**Table 1:** Demographic information of the study participants

	TBM (*n* = 41)	Non-TBM (*n* = 6)	
Sex, female/male	13/28	4/2	
Age in years:			
Mean (range)	17.4 (0.3–57)	39.96 (0.0001–53)	
Median (IQR)	17.8 (2.3–26.3)	0.92 (0.01–19.95)	
TBM status, n (%):			
TBM diagnosis (definitive)	40 (98)	No	
TBM stage on admission:			
Stage I	3 (7.3)	NA	
Stage IIa	7 (17.1)	NA	
Stage IIb	5 (12.2)	NA	
Stage III	21 (51.2)	NA	
Not reported	5 (12.2)	NA	
Last known TBM stage before death:			
Stage I	3 (7.3)	NA	
Stage IIa	1 (2.4)	NA	
Stage IIb	0 (0)	NA	
Stage III	25 (61)	NA	
Not reported	12 (29.3)	NA	
Concurrent miliary TB, n (%)	21 (51.2)	NA	

*IQR*  interquartile range

### Chemicals and standards

The internal standard used in the analysis was 3-phenylbutyric acid (Snow, 2024). Methanol (Honeywell International Inc., Muskegon, Michigan, USA; CAS # 67-56-1), Milli-Q water, and dichloromethane (Sigma-Aldrich, St. Louis, Missouri, USA; CAS # 75-09-2) served as solvents. The derivatization reagents were N, O-bis(trimethylsilyl)trifluoroacetamide (BSTFA) and methoxyamine hydrochloride (MOX-HCl) (Sigma-Aldrich, St. Louis, Missouri, USA). To deparaffinize the samples, xylene (Sigma-Aldrich, St. Louis, Missouri, USA; CAS # 1330-20-7) was used. Additional information on the chemicals and standards used can be found in our protocol paper (Isaiah et al., 2024).

### Sample analysis

#### Laser microdissection

**Fig. 1:**
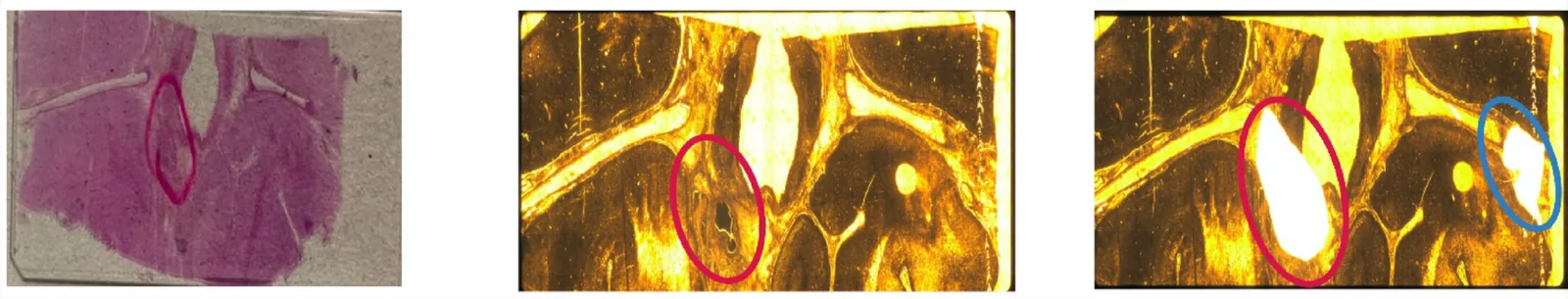
Example of sample selection by laser microdissection. Left: H&E-stained region of brain tissue containing a well-defined granuloma (outlined in red). Middle: the same block scanned with the laser microdissection microscope (×4 magnification) to identify areas for dissection. Right: remaining brain tissue after dissection of two areas—granulomatous tissue (outlined in red) and surrounding tissue (outlined in blue)

TBM blocks were sectioned into 20-micron-thick slices with a microtome, and each slice was placed on a membrane slide and a histology slide (for H&E staining). An experienced pathologist identified and marked granuloma regions on the H&E slides, ensuring they were well-developed and ready for laser microdissection on the corresponding membrane slides. The Molecular Machines and Industries (MMI) CellCut laser microdissection system was used to microdissect the granuloma regions (Fig. 1). Samples were collected using MMI isolation caps and then transferred to a labeled 2 mL microcentrifuge tube for further analysis. Control blocks were sectioned at 20 μm with a microtome and carefully transferred into labeled 2 mL microcentrifuge tubes using forceps.

### Metabolite extraction

The FFPE brain tissue samples were first heated at 70 °C for 1 min (to melt the wax and reduce the risk of contaminants during metabolite extraction). The samples were homogenized at 30 Hz for 5 min and deparaffinized by two 1 mL xylene washes, followed by centrifugation for 5 min at 20,000x*g* at 4 °C after each xylene wash. The supernatants were discarded, and the tissue was weighed (~ 20 mg). An internal standard (3-phenylbutyric acid; 50 ppm) was added to the samples (50 µL). Quality control (QC) samples were pooled from non-TBM controls, deparaffinized, homogenized (in xylene), weighed (~ 20 mg), and aliquoted for each batch. Each QC sample was prepared and analyzed in the same manner as the other samples. Detailed information on the extraction and derivatization can be found in our published protocol (Isaiah et al., 2024). Metabolites were extracted in two parts: polar and apolar. The polar metabolites were extracted with 200 µL of a methanol-water (1:1, v/v) mixture. Samples were homogenized at 30 Hz, vortexed, sonicated at high frequency without heat, and centrifuged at 20,000 x g at 4 °C for 5 min. The supernatant was dispensed into a GC-MS vial and stored on ice. Thereafter, an apolar extraction with 250 µL of a dichloromethane-methanol (3:1) mixture was performed on the pellet, following the procedure for the polar solvents above. The supernatants from the polar and apolar extractions were combined in a GC-MS vial. The combined supernatants were dried using a SpeedVac and derivatized using a two-step procedure: oximation followed by silylation. The oximation step involved adding 20 µL of MOX-HCl dissolved in pyridine (20 mg/mL) to the dried samples, vortexing for 5 min, and incubating at 37 °C for 90 min. The silylation step involved adding 40 µL of BSTFA with 1% v/v trimethylchlorosilane (TMCS), followed by incubation at 37 °C for 60 min. Derivatized samples were transferred to spring-bottom vial inserts, capped, and placed in an Agilent autosampler for GCxGC-TOFMS analysis.

### Experimental design and GCxGC-TOFMS analysis

Samples were loaded in a randomized order, with QC samples at the beginning, middle, and end of each batch. QC samples were also run repeatedly at the start of each batch to condition the GC columns. An average of 19 samples was run per batch. One microliter of each derivatized sample (including blanks which was made up of all the extraction solvents without the tissue sample) was injected at an optimized 1:5 split ratio on a LECO Pegasus 4D GCxGC-TOFMS fitted with an Agilent 7890 GC system and TOF-MS from LECO Africa, equipped with a Restek Rxi-5Sil primary column (dimensions: 28.2 m length, 250 μm diameter; 0.25 μm film thickness) and a Restek Rxi-17 secondary capillary column (dimensions: 1.3 m length; 250 μm diameter; 0.25 μm film thickness) for chromatographic separation. Helium was used as the carrier gas at a constant flow of 1.4 mL/min. Septum purge flow was set to 3 mL/min, and the gas saver flow was set to 15 mL/min. The front inlet temperature was set to 250 °C throughout the run. The primary oven was initially set to 70 °C for 1 min, then increased at 3 °C/min to 325 °C, and maintained at 325 °C for 3 min after the run to clean the GC columns. The secondary oven was set to a temperature offset of + 5 °C, and the modulator was enabled to 15 °C at 3 s, with a 0.7 s pulse interval. The chiller was set to − 80 °C, and the transfer line temperature was set to 225 °C (Isaiah et al., 2024). A total of eight batches, each with 19 samples, were run over eight successive days.

The ChromaTOF software (version 4.72.0.0; LECO, St. Joseph, MI, USA) was used to analyze the data. Smoothing parameters in the software were applied for baseline subtraction, peak detection, and deconvolution. The expected peak width was set to at least 3 s, and the signal-to-noise ratio was set to 100. Peaks were annotated using in-house and National Institute of Standards and Technology (NIST) spectral libraries. Compounds were identified based on a 70% spectral match. An additional manual confirmation of the identified compound was performed by visually comparing its mass spectral fragment pattern to the library and to the standard’s expected retention time index. All identified metabolites are putative and conform to the Metabolomics Standards Initiative (MSI) level 2.

### Data analysis

Raw GCxGC-TOFMS data were preprocessed using a standardized metabolomics protocol, and each variable was normalized to the internal standard (3-phenylbutyric acid). A 50% filter was applied, removing compounds detected in fewer than 50% of samples within each group. MetaboAnalyst 6.0 (www.metaboanalyst.ca/) was used for all statistical analyses, and data were log10-transformed and Pareto-scaled. To assess batch effects, QC samples were used. Unsupervised principal component analysis (PCA) was used to visually compare sample groups and assess clustering and separation between them (TBM and non-TBM controls). Statistical significance was based on multivariate supervised partial least squares-discriminant analysis (PLS-DA) variable importance in projection (VIP) values > 1.0, and univariate measures with fold change > 2.0, *t*-test (FDR *p*-value < 0.05), and Cohen’s effect size (> 0.8) (Du Preez & Loots, 2013). A heatmap and a debiased sparse partial correlation (DSPC) network were used to correlate significant metabolites with patient clinical information.

## Results

The quality of the metabolomics data can be evaluated by examining the PCA score plot of the experimental groups alongside the QC samples (Fig. S1). Because the pooled QC samples comprised only aliquots of the control samples (due to limited sample volumes in the TBM group), the QC group shows a distribution similar to that of the control samples. No discernible pattern or drift was observed in the QC samples; hence, there was no drift across the eight batches analyzed. Based on this quality-assurance data (Fig. S1), we conclude that the observed separation between the TBM and non-TBM groups is consistent with underlying biological differences following QC-based filtering, although a contribution from residual analytical variability cannot be completely excluded.

**Fig. 2:**
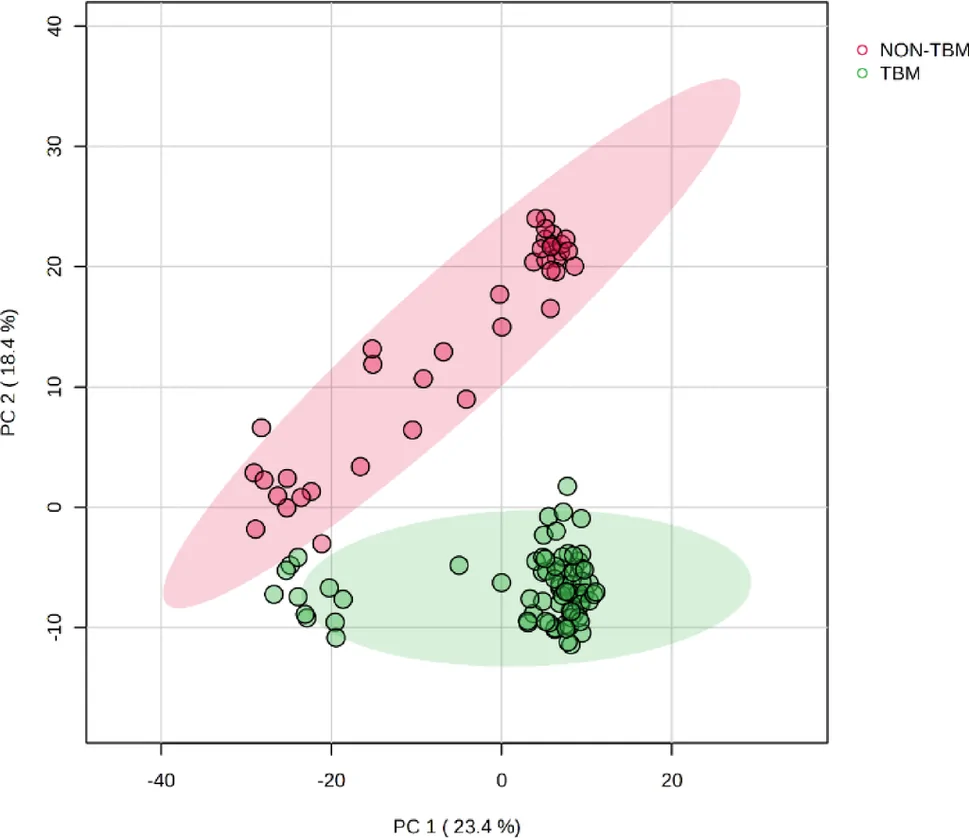
A PCA scores plot shows clustering within the sample groups, albeit with some heterogeneity. The red cluster represents the non-TBM group, and the green cluster represents the TBM group. The two groups are clearly and naturally separated. The first two principal components (PC1 and PC2) explain 23.4% and 18.4% of the total variance, respectively, accounting for 40.8% of the overall variation

**Fig. 3:**
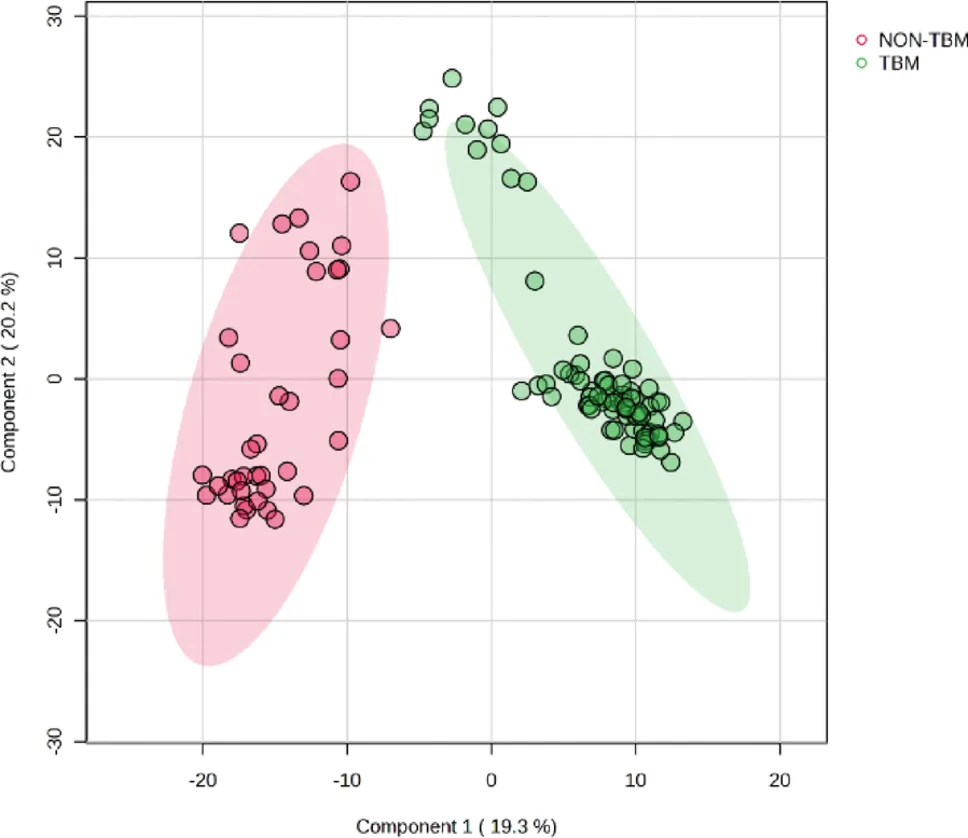
A PLS-DA scores plot shows clear discrimination between the non-TBM (red) and TBM (green) groups along components 1 and 2. PLS-DA model performance was evaluated using cross-validation, yielding R^2^ and Q^2^ of 0.89 and 0.87, respectively, indicating strong predictive ability. Permutation testing confirmed that the observed class separation was not due to overfitting (*p* < 0.05)

Figure 2 is an unsupervised PCA score plot comparing metabolite data from TBM and non-TBM brain tissue, showing clear clustering and separation between the two groups and indicating statistically significant differences in their underlying metabolic profiles. Figure 3 is a supervised PLS-DA score plot that clearly discriminates between the two experimental groups. The PLS-DA validation values (Q^2^ and R^2^) were close to 1 for components 1 (0.87) and 2 (0.89), respectively, with a prediction accuracy of 0.978. The PLS-DA plots did not overfit the data (permutation test *p*-value = 0.05) and revealed that 294 of the 454 detected metabolites were responsible for the significant separation (VIP > 1.0) observed between the groups.

A total of 41 metabolites were statistically significant (VIP > 1.0, fold change > 2.0, *t*-test FDR *p*-value < 0.05, and effect size > 0.8) in differentiating the TBM group from the non-TBM group. Table 2 lists the 41 significant metabolites, grouped by metabolite class. The relative concentrations were calculated using normalized abundance values, and the multivariate analyses were performed after the data were log10-transformed and Pareto-scaled in MetaboAnalyst. Of these 41 metabolites, ten, namely (2Z,5Z)-2, 5-pentadecadien-1-ol, arachidonic acid, D-chiro-inositol, 4-ketoglucose, hexanol, methyl heneicosanoate, pimelic acid, pipecolic acid, pregnenolone, and L-pyroglutamic acid, were significantly reduced to concentrations below the GCxGC-TOFMS detection limit across all TBM cases.

**Fig. 4:**
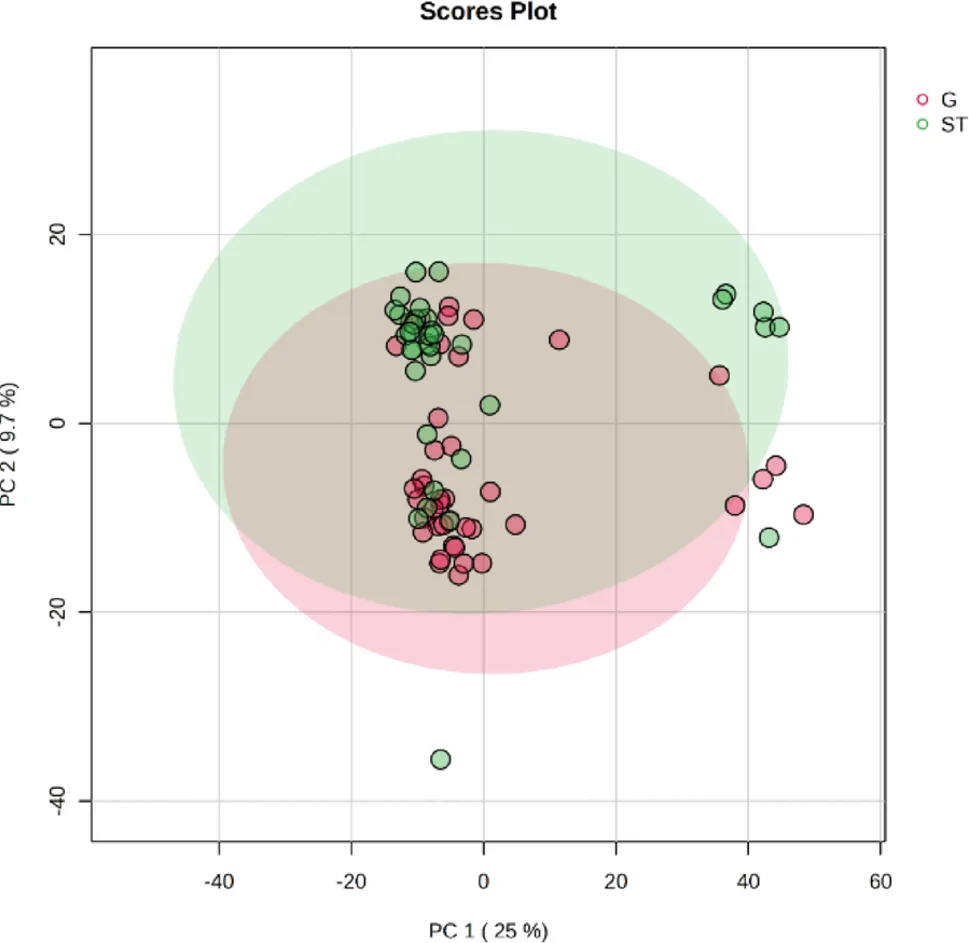
A PCA scores plot shows clustering within the sample groups. The red cluster indicates the granuloma region (G), while the green cluster indicates the surrounding tissue (ST) group. There is no clear, natural separation between the two groups. The first two principal components (PC1 and PC2) explain 25% and 9.7% of the total variance, respectively, together accounting for 34.7% of the overall variation in the data

Comparisons were made between the granuloma region and the surrounding tissue (Fig. 4), and 6 metabolites (Table 3) were identified as statistically significant using univariate tests with fold changes > 2.0 and *t*-test FDR *p*-values < 0.05, and multivariate tests with VIP scores > 1.0. These metabolites (tritriacontane, tricosane, 17-pentatriacontene, tetracosane, 3-tridecene, and 5-eicosene) are all classified as hydrocarbons (alkanes and alkenes). Both 3-tridecene and 5-eicosene were significantly upregulated in the granuloma regions, whereas the other four metabolites were significantly downregulated in the surrounding tissue. However, the effect sizes were small (0.2–0.34) across all 6 metabolites, indicating low practical significance.

### Linking significant metabolites to patient clinical information

**Fig. 5:**
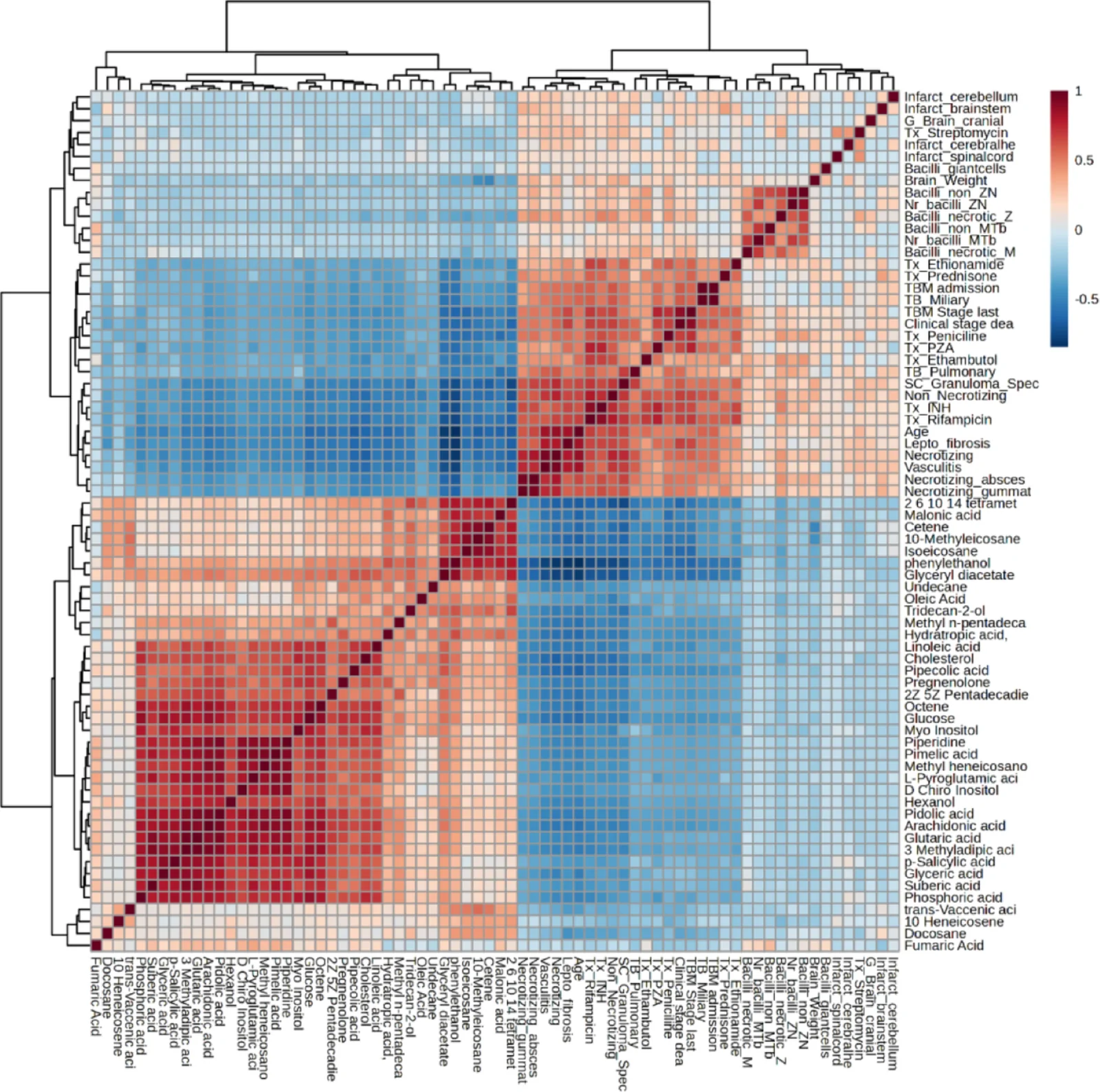
Heatmap shows the correlation between significant metabolites and patients’ clinical states. Overall, significantly altered metabolites appear to positively correlate with clinical data

We further analyzed the significant metabolites in relation to the patients’ clinical data (Fig. 5). We found that fumaric acid correlated positively with *Mtb* bacilli present in the necrotic area (*r* = 0.16), *Mtb* bacillary load (*r* = 0.21), *Mtb* bacilli present in the non-necrotic area (*r* = 0.30), brain weight in grams (*r* = 0.15), and infarction in the cerebellum (*r* = 0.003) (see Table 4 for definitions to clinical terms). Docosane also showed positive correlations with granulomas in the brain/infratentorial/cranial nerve roots (*r* = 0.03), infarction in the brainstem (*r* = 0.17), and infarction in the cerebellum (*r* = 0.03). 10-Heneicosane correlated positively with infarction in the spinal cord (*r* = 0.08), trans-vaccenic acid correlated positively with infarction in the cerebral hemisphere (*r* = 0.02), and phosphoric acid correlated positively with infarction in the cerebral hemisphere (*r* = 0.099) and with prescribed therapy (streptomycin) during admission (*r* = 0.05). Suberic acid correlated positively with *Mtb* bacilli present in the necrotic area (*r* = 0.07), glyceric acid correlated positively with *Mtb* bacilli present in the necrotic area (*r* = 0.015), and p-salicylic acid correlated positively with infarction in the spinal cord (*r* = 0.07) and infarction in the cerebral hemisphere (*r* = 0.11). Tridecan-2-ol correlated positively with infarction in the spinal cord (*r* = 0.06), oleic acid correlated positively with infarction in the spinal cord (*r* = 0.05), and with infarction in the cerebral hemisphere (*r* = 0.03). Glyceryl diacetate 2-linolenate correlated positively with infarction in the spinal cord (*r* = 0.003), isoeicosane correlated positively with infarction in the cerebellum (*r* = 0.04), 10-methyleicosane correlated positively with infarction in the cerebellum (*r* = 0.03), and cetene correlated positively with infarction in the brainstem (*r* = 0.05) and with infarction in the cerebellum (*r* = 0.03).

**Fig. 6:**
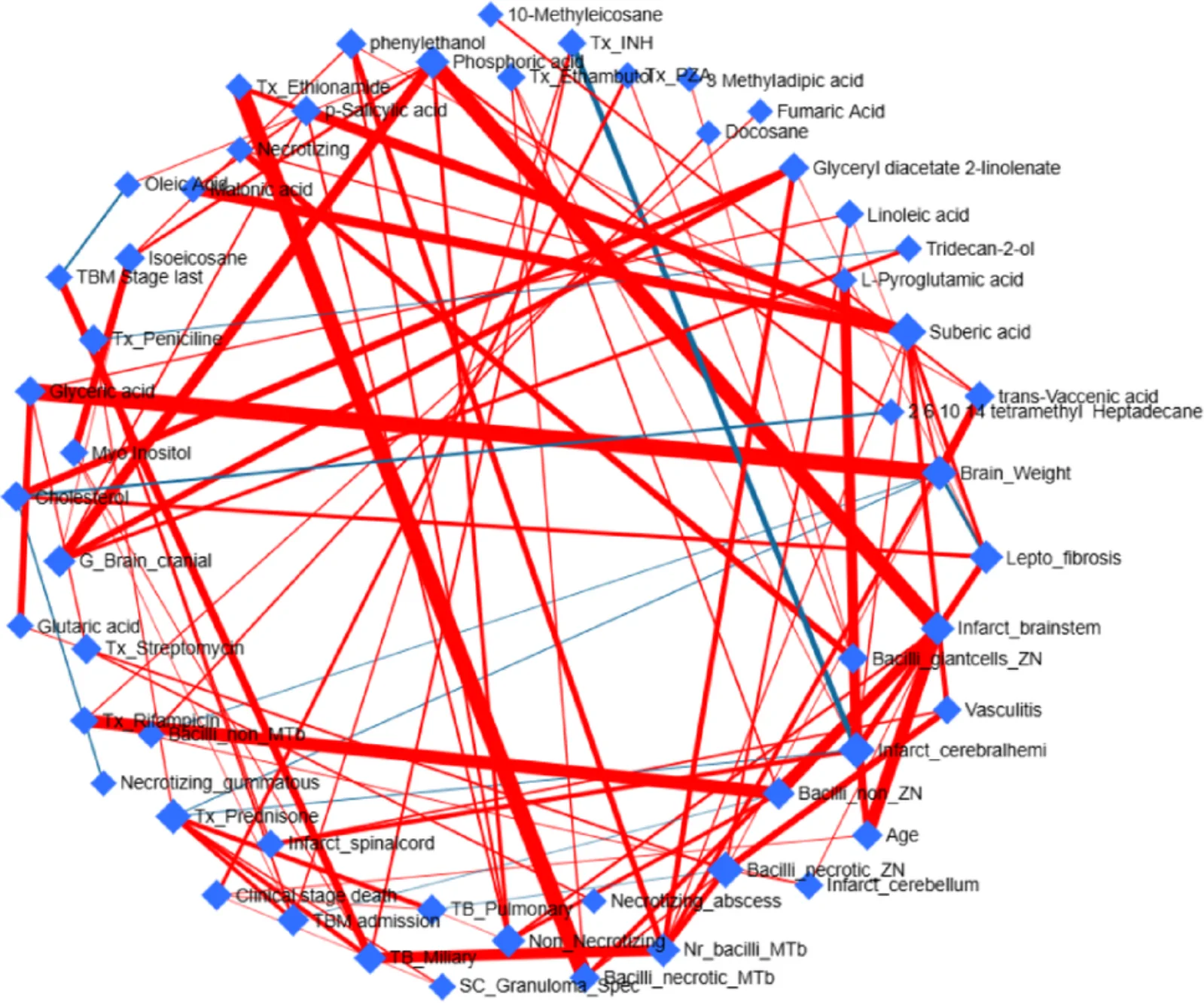
Debiased sparse partial correlation (DSPC) network (Basu et al., 2017) showing associations between significant metabolites and the patient’s clinical information. The nodes represent metabolites and clinical information, and the edges represent association measures. The red lines indicate positive correlations, while the blue lines indicate negative correlations. The line intensity indicates significance

The DSPC network analysis on MetaboAnalyst (Fig. 6) showed that metabolites such as 2-phenylethanol were positively associated with infarcts in the brainstem and with granulomas in the brain/infratentorial/cranial nerve roots. The granulomas in the brain/infratentorial/cranial nerve roots were also positively associated with glyceryl diacetate-2-linolenate. Suberic acid had significant positive associations with ethionamide administered on admission. In contrast, the final TBM staging before discharge or death had a negative association with oleic acid and a significant positive association with miliary TB. Other metabolites were correlated with one another and were not directly associated with other clinical information.

**Table 2:** Forty-one metabolites that are statistically significant between TBM and Non-TBM samples based on the following cutoff criteria: fold change > 2.0, *t*-test FDR *p*-value < 0.05, effect size > 0.8, and PLS-DA VIP value > 1.0. SD = standard deviation

Metabolite name (PubChem/ChemSpider ID)	Non-TBM	TBM	Log_2_ fold change	FDR	PLS-DA	Effect size	
	Relative metabolite concentration (mg/g tissue) ± SD	Relative metabolite concentration (mg/g tissue) ± SD	[Non-TBM/TBM]	*p*-value	VIP value	*d*-value	
Hydrocarbons (alkanes and alkenes)							
Undecane (13619)	12.92 ± 0.065	0.05 ± 0.0000052	8.75	3.95E-11	1.25	5.72	
Octene (7833)	1.57 ± 0.00028	0.10 ± 0.000035	4.84	4.30E-17	1.53	53.27	
2,6,10,14-Tetramethylheptadecane (27012)	254.45 ± 5.32	7.20 ± 0.0103	1.50	1.01E-21	2.33	4.73	
(5E)-5-Eicosene (4516747)	328.18 ± 7.38	12.26 ± 0.041	5.24	5.30E-23	2.42	4.60	
(10E)-10-Heneicosene (4516702)	229.77 ± 12.3	20.91 ± 0.072	3.85	2.18E-04	0.96	9.35	
(E)-3-Tridecene (4515229)	469.95 ± 56	47.99 ± 1.04	4.19	1.23E-22	2.52	5.60	
Cetene (11889)	107.723 ± 101.17	121.89 ± 3.02	2.97	4.14E-09	1.04	4.31	
10-Methyleicosane (37607)	70682.43 ± 319738.7	793.35 ± 47	4.12	9.26E-10	1.24	1.69	
2-Methylnonadecane (120790)	17431.27 ± 19126.89	2211.84 ± 435	2.40	5.15E-06	1.14	2.16	
Docosane (11899)	95395.31 ± 1125102.86	11556.51 ± 34050.012	4.18	1.48E-3	3.78	1.74	
Alcohols							
(2Z,5Z)-2, 5-Pentadecadien-1-ol (5364952)	1.79 ± 0.00068	0 ± 0	10.20	2.48E-15	1.26	2.29	
Hexanol (HMDB0012971)	0.68 ± 0.000083	0 ± 0	11.93	2.43E-12	1.14	0.95	
2-Phenylethanol (5830)	197.04 ± 13.6	0.02 ± 0.0000014	13.99	5.39E-74	2.99	1.615	
(6R,10R)-6,10,14-Trimethyl-2-pentadecanol(530418)	6.08 ± 0.0017	0.27 ± 0.000033	4.8	1.31E-08	1.09	34.16	
Undecanol (7892)	19.67 ± 0.15	0.65 ± 0.00057	5.81	2.52E-18	1.76	13.15	
Tridecan-2-ol (14701)	72.01 ± 0.89	2.58 ± 0.0066	3.93	2.86E-13	1.79	7.81	
Amino acids							
Pipecolic acid (826)	30.40 ± 0.31	0 ± 0	17.06	9.90E-22	2.00	1.66	
L-Pyroglutamic acid (7127)	5.62 ± 0.0026	0 ± 0	14.63	2.87E-14	1.47	0.8	
Fatty acids and lipids							
Arachidonic acid (392692)	5.29 ± 0.00045	0 ± 0	13.30	1.74E-16	1.47	0.85	
Methyl heneicosanoate (21054)	8.17 ± 0.0016	0 ± 0	13.77	2.04E-11	1.18	1.15	
Methyl pentadecanoate (21988)	7.38 ± 0.012	0 ± 0	2.48	8.31E-14	1.33	2.62	
Pregnenolone (8611)	29.08 ± 0.082	0 ± 0	10.65	5.66E-13	1.39	2.19	
Pimelic acid (376)	2.57 ± 0.00079	0 ± 0	13.20	2.67E-12	1.20	1.22	
3-Methyl adipic acid	0.32 ± 0.00086	0.0212 ± 0.000000044	6.98	6.73E-12	1.34	13.33	
Suberic acid (10025)	0.69 ± 0.000056	0.02 ± 0.00000022	5.65	1.51E-09	1.00	65.69	
2-Methylhexanedioic acid (455411)	3.25 ± 0.00086	0.02 ± 0.00000044	6.97	6.73E-12	1.34	13.32	
Malonic acid (844)	121.34 ± 7.54	3.47 ± 0.018	5.92	6.87E-19	2.21	4.09	
Glyceryl diacetate 2-linolenate (21159744)	131.82 ± 0.59	0.08 ± 0.000000815	11.61	1.08E-46	2.59	2.05	
p-Salicylic acid (24532634)	2.91 ± 0.00059	0.15 ± 0.000005	4.82	1.07E-06	1.10	37.87	
Linoleic acid (4444105)	11.58 ± 0.015	0.25 ± 0.000035	6.39	3.64E-17	1.61	17.45	
Cholesterol (5775)	96.86 ± 0.243	0.58 ± 0.000013	7.28	2.86E-13	1.74	3.86	
Oleic Acid (393217)	204.50 ± 3.33	5.252 ± 0.0097	4.47	4.81E-08	1.46	4.83	
trans-Vaccenic acid (4444571)	2905.39 ± 849.845	358.49 ± 31.9	3.21	1.64E-06	1.55	3.67	
Organic acids							
Fumaric acid (10197150)	3.19 ± 0.0015	0.06 ± 0.0000072	6.73	2.64E-13	1.43	18.76	
Glyceric acid (732)	4.83 ± 0.004	0.107 ± 0.000011	5.92	2.36E-09	1.32	16.44	
Glutaric acid (723)	1.23 ± 0.00018	0.28 ± 0.0000021	5.51	5.43E-12	1.25	43.35	
Sugars							
Glucose (9601862)	9.39 ± 0.0011	0 ± 0	8.24	2.29E-17	1.51	1.29	
D-chiro-Inositol (10254647)	7.74 ± 0.015	0 ± 0	14.61	4.44E-20	1.64	2.00	
Myo Inositol (10239179)	312.57 ± 6.78	0.23 ± 0.0000038	10.41	6.50E-10	1.73	1.15	
Inorganic compounds							
Phosphoric acid (979)	63.21 ± 2.3164	0.02 ± 0.00000051	12.94	6.35E-09	1.59	1.30	
Organic compounds							
Piperidine	0 ± 0	0.71 ± 0.0099	15.56	2.32E-12	1.41	0.84	

*FDR* false discovery rate

**Table 3:** Six statistically significant metabolites between the granuloma region and the surrounding tissue based on cutoff criteria: fold change > 2.0, *t*-test FDR *p*-value < 0.05, and PLS-DA VIP value > 1.0

Metabolite name (ChemSpider ID)	Log_2_ fold change [G/ST]	FDR *p*-value	VIP-value	Effect size
Tritriacontane (11905)	− 4.075	0.0017	4.10	0.02
Tricosane (12017)	− 1.92	0.0017	3.53	0.02
17-Pentatriacontene (HMDB0302842)	− 3.13	0.0023	3.92	0.03
Tetracosane (12072)	− 2.66	0.022	3.16	0.03
3-Tridecene (4515229)	9.09	0.024	2.029	− 0.35
5-Eicosene (17879)	1.44	0.038	1.90	− 0.05

*FDR* false discovery rate

Table 3 lists the significant metabolites when comparing the granuloma region to the surrounding tissue. Four metabolites, namely tritriacontane, tricosane, 17-pentatriacontene, and tetracosane, were significantly downregulated, while two metabolites—3-tridecene and 5-eicosene—were significantly upregulated in the granuloma region. The effect sizes were small (low practical significance) for all six metabolites in Table 3.

**Table 4:** Definitions for clinical terms

Clinical term	Definition
Necrotic area	A localized part of the tissue that has died due to *Mtb* infection.
Non-necrotic area	Area of tissue that is still viable.
Bacilli load of *Mtb*	The quantity of *Mtb* present in the sectioned sample when viewed under a microscope.
Infarct	Inadequate blood supply (ischemia/hypoxia) leading to tissue necrosis.
Granuloma	Localized cluster of immune cells – histopathological hallmark of tuberculosis (TB).
TBM stage 1	The initial prodromal phase is characterized by nonspecific, mild symptoms that last 1–3 weeks. Glasgow Coma Score (GCS) = 15.
TBM stage 2	Moderate, intermediate phase characterized by drowsiness, confusion, or early focal neurological deficits (e.g., cranial nerve palsies, hemiparesis). GCS = 11–14.
TBM stage 3	The most advanced and severe stage of *Mtb* infection in the brain, characterized by coma, delirium, severe neurological deficits (such as paralysis), and multiple cranial nerve palsies. GCS < 11.
Infratentorial/cranial nerve roots	Nerves that emerge from the brainstem within the posterior cranial fossa, located below the tentorium cerebelli.
Brainstem	The stalk-like posterior part of the brain, connecting the cerebrum to the spinal cord, is composed of the midbrain, pons, and medulla oblongata.
Cerebellum	A major brain structure located at the back of the skull, below the cerebrum and behind the brainstem
Cerebrum	The largest, topmost part of the brain.

## Discussion

The human brain is composed mainly of water and lipids, with other chemical compounds such as carbohydrates, proteins, and salts present in small amounts. The blood-brain barrier selectively protects the brain from invasion by pathogenic organisms and toxins. Under normal conditions, brain homeostasis is well-regulated; however, prolonged or chronic disturbances may disrupt normal blood-brain barrier function and brain metabolism, triggering a cascade of immune responses (Roh et al., 2016, Müller et al., 2025). It remains unclear how *Mtb* invades the brain despite the brain’s defense mechanisms. However, it is known that *Mtb* causes prolonged effects even with low microbial presence, which, when left untreated, could lead to irreparable damage in the brain. Despite the brain’s defense system, *Mtb* survives. To understand the pathophysiology of TBM, the metabolic footprint and survival tactics of *Mtb* must be elucidated. Some of the observed mechanisms will be explained in relation to the metabolites significantly altered in advanced TBM cases.

### *Mtb* possibly breaks down hydrocarbons to produce energy

**Fig. 7:**
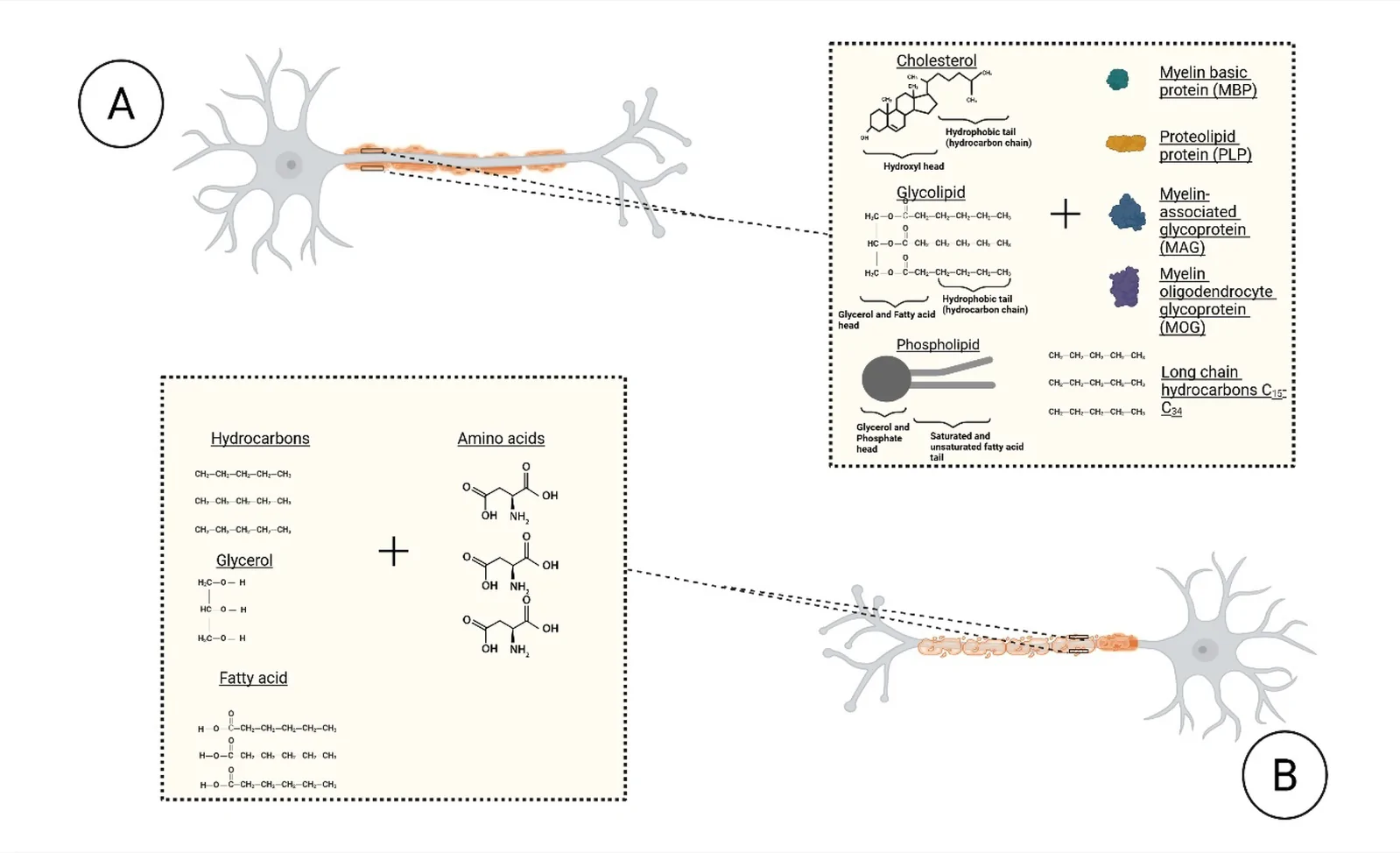
Authors’ illustrative depiction of a neuron with **A** a healthy myelin sheath and its associated proteins and metabolites, and **B** a degenerating myelin sheath and degraded metabolites

The degeneration of the myelin sheath, also known as demyelination (Fig. 7), typically occurs in severe neurological disorders, such as multiple sclerosis, and has been linked to a range of health complications, including autoimmune disorders, inflammation, and infections. These disorders cause hypoxia and ischemia (Ohno & Ikenaka, 2019, Hollingworth et al., 2025). After demyelination, remyelination can restore normal neurological function; however, it occurs only when neuronal damage is minimal and reversible. Metabolically, we observed that hydrocarbons (alkanes and alkenes), namely (10E)-10-heneicosene, (5E)-5-eicosene, (E)-3-tridecene, 10-methyleicosane, 2,6,10,14-tetramethylheptadecane, cetene, octene, and undecane, were either depleted or absent in TBM groups compared with non-TBM groups. The significant reduction in these hydrocarbons may reflect altered host lipid metabolism, tissue injury, or potential utilization by *Mtb*; however, the present study does not directly distinguish among these possibilities using histological assessment of myelin integrity. Metabolomics is an evolving field and may suggest mechanisms not initially thought. Furthermore, these complex hydrocarbons may be catabolized to serve as a source of free carbon in the body (Coelho et al., 2013, Cooper & Adams, 2022). *Mtb* has been shown to utilize these chemical compounds for energy and survival (Bourre et al., 1977, Isaiah et al., 2025). Similarly, the predominant class distinguishing the granuloma region from the surrounding tissue, although not confirmed by the statistical analyses (weak correlations), is hydrocarbons, which may suggest the importance of this class of compounds in TBM disease and should be investigated further in studies integrating metabolomic, lipidomic, and histological approaches (Table 3).

### *Mtb* possibly utilizes fatty acids and lipids as protective shields and as an energy source

It was observed that the lipids / fatty acids and related compounds, such as arachidonic acid, glyceryl diacetate-2-linolenate, cholesterol, linoleic acid, methyl heneicosanoate, methyl pentadecanoate, and oleic acid extracted from the FFPE tissue in the TBM group, were significantly reduced when compared to the non-TBM controls. This can be explained by *Mtb* actively using these fatty acids and lipids as protective shields against antibiotics and the body’s immune system, since its waxy cell wall is composed mostly of lipids (Batt et al., 2020, Marrakchi et al., 2014). This fatty acid was also negatively correlated with final TBM staging at last discharge or death (Fig. 6), indicating that the higher the disease severity, the less likely oleic acid was present. Arachidonic acid is an important fatty acid in inflammation, as it is released in response to inflammation by phospholipase A2. It is said to trigger the production of other inflammatory metabolites through the actions of eicosanoids and is a major indicator of cell wall damage, thereby increasing blood-brain barrier permeability (Higgins & Lees, 1984, Wang et al., 2007). Linoleic acid, a primary form of arachidonic acid, has important membrane protective properties and is a precursor of eicosanoids (Mori & Hodgson, 2013). These metabolites are significantly lower in TBM, suggesting increased *Mtb* activity against them in severe disease. Pregnenolone, a steroid hormone synthesized from cholesterol (Lin et al., 2022), has been found to maintain homeostasis during inflammation by suppressing activated macrophages (Murugan et al., 2019). This was also below the detection limit in TBM cases. Oleic acid is an important metabolite with neuroprotective properties essential for myelin sheath synthesis; however, in TBM, it creates an acidic environment conducive to *Mtb* proliferation (Song et al., 2019, Gouzy et al., 2021). Cholesterol is important for the formation of the myelin sheath, which is important for brain function. The brain should have the highest concentration of cholesterol; however, in our results (Table 2), the non-TBM samples had a cholesterol concentration of 96.86 ± 0.24 mg/g of brain tissue, while the TBM samples had over a hundred-fold lower cholesterol concentration (0.58 ± 0.000013 mg/g of brain tissue). This is suggestive of the detrimental effect of *Mtb* on the human brain, as it could be using nearly all of the brain’s cholesterol for its survival (Shin et al., 2024, Pandey & Sassetti, 2008). Trans-vaccenic acid is an important fatty acid derived from dairy foods and certain meats, and it is important in neurological health and known to promote anti-tumor immunity as an immunomodulator. *Mtb* is known to use this diet-derived fatty acid as its source of nutrition, possibly explaining the significantly low levels observed in TBM samples (Sanjulian et al., 2024, Fan et al., 2023, Laval et al., 2021). Suberic acid and pimelic acid (fatty acids) were also significantly reduced in TBM cases in this study. These results contrast with a study that found elevated levels of these fatty acids in urine from TBM cases (Isaiah et al., 2024). This could be because these fatty acids are completely consumed in the brain tissue under fatal TBM conditions (i.e., extreme endpoint). The reduced abundance of malonic acid, synthesized from malonyl-CoA, is an important indicator of neurodegeneration as seen in diseases such as Alzheimer’s and Parkinson’s disease. Although required in minute amounts, malonic acid has neuroprotective and anti-inflammatory properties. It is involved in the synthesis of fatty acids, suggesting that the host is trying to compensate for fatty acids lost to *Mtb* by synthesizing more fatty acids (Lee et al., 2021). In addition to using fatty acids and lipids as nutrient sources and for survival, *Mtb* can adapt to the most unfavorable environments for Mycobacteria spp.

### The ability of *Mtb* to survive in hypoxic environments

*Mtb* can survive in oxygen-deprived environments due to its high resilience without replicating (Berney & Cook, 2010). An alternative hypothesis is that the loss of oxygen (ischemia) experienced during the terminal stages of TBM may be due to *Mtb*’s ability to scavenge oxygen in hypoxic situations for survival, and for it to remain dormant when there is a complete shutdown of oxygen (Berney & Cook, 2010, Kalia et al., 2023). *Mtb*, when studied under hypoxic situations, only survives and does not replicate because it requires oxygen for replication (Berney & Cook, 2010, Reichlen et al., 2017). But in the case of TB granuloma (severely hypoxic), *Mtb* can replicate without limit (Cooper et al., 2024, Lyu et al., 2024). This suggests that there may be an alternative source of oxygen outside the environment. Chances are that rather than using fatty acids and lipids as its only energy source, *Mtb* would scavenge oxygen from some of these fatty acids [the same way it remodels lipids for its phosphorus needs (Gray et al., 2025)], transfer them to the hydrocarbons and use these as an additional energy source and for replication. Hence, some studies have found hydroxylated fatty acids in cases of TB from CSF and urine samples (Isaiah et al., 2024, Le et al., 2025, Churchill et al., 1999). In our study, we did not detect hydroxylated fatty acids in post-mortem brain tissue from TBM cases; instead, we detected methylated fatty acids (methyl heneicosanoic, methyl pentadecanoic, and methyl hexanedioic acids). This may be because of the type of sample analyzed and derivatized (brain tissue). Further work is needed to confirm this hypothesis.

**Fig. 8:**
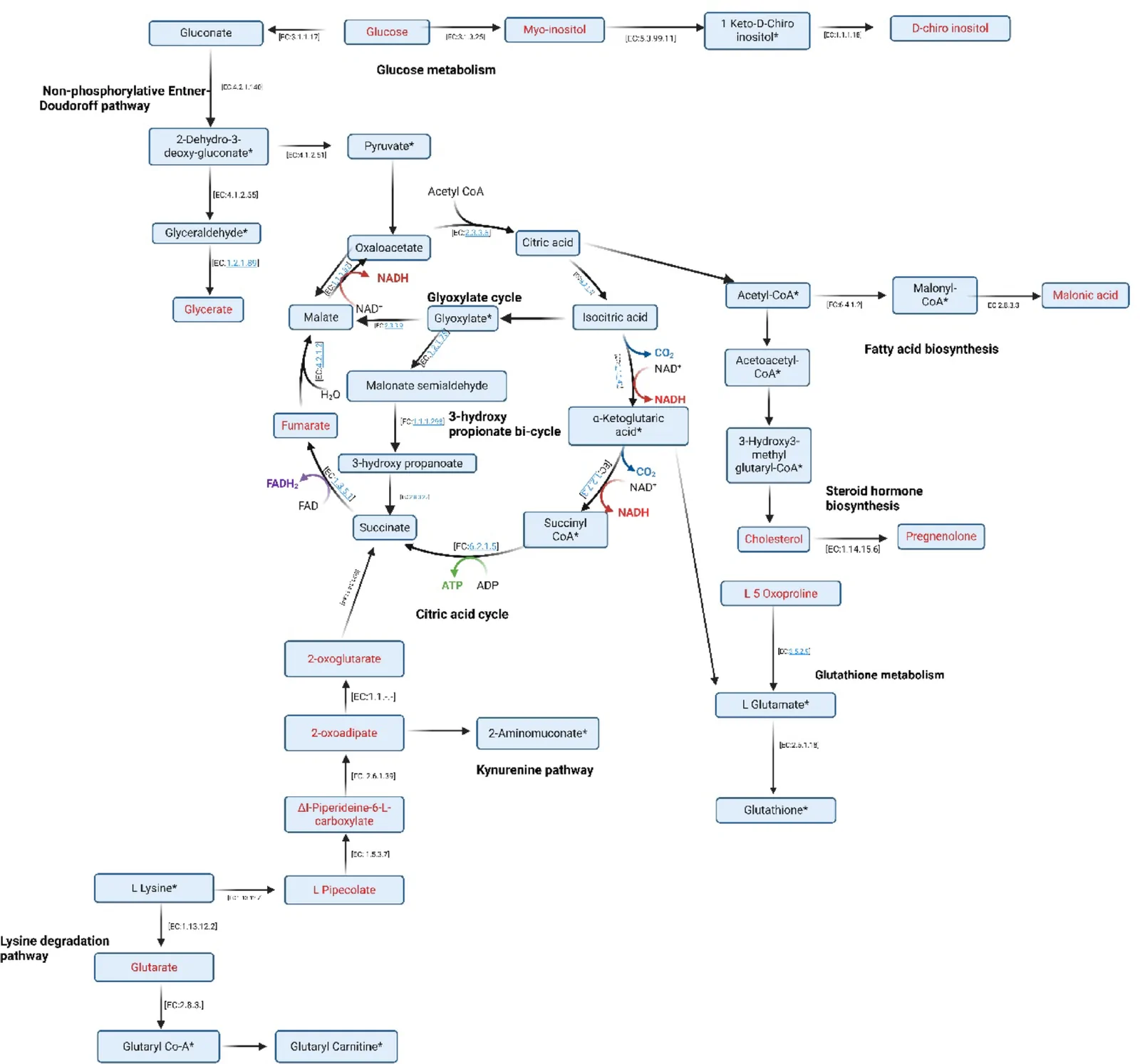
Pathway showing the metabolites that differentiate the TBM group from the non-TBM group, with significant metabolites in red. The asterisk (*) indicates metabolites not detected in the dataset but that are part of the pathway. Some of the compounds depicted in the pathway were in their modified forms in the dataset (the actual compound may be short of oxygen and, in the pathway, exists in its oxidized state)

### *Mtb* uses organic acids for metabolism and growth

Organic acids such as fumaric acid, glyceric acid, and glutaric acid are important energy sources for the host and are largely metabolized through the citric acid cycle (Fig. 8). A significant shortage of these organic acids indicates severe energy depletion in the host and may be exploited by *Mtb* (Pacífico et al., 2018). Glutaric acid is synthesized from glutaryl-CoA and is elevated in individuals with multiple acyl-CoA dehydrogenase deficiency (MADD), a condition characterized by low glucose and high ammonia levels in the blood, which could signify excessive breakdown of the body’s fat for energy production (Mason et al., 2023, Chang et al., 2024). Previous studies on CSF have shown that most organic acids, such as glutamate, citrate, and pyruvate, were depleted in TBM cases; however, fumaric acid and glyceric acid were not reported (Zyl et al., 2020, Mason et al., 2015). Fumaric acid showed a positive correlation (although statistically weak) with bacilli in the non-necrotic area of *Mtb*-infected samples (Fig. 8). These findings are exploratory and suggest potential for detecting *Mtb*, as fumaric acid is known to be important in preventing biofilm formation in bacilli. The positive association could mean that reduced fumaric acid levels were observed in patients with fewer *Mtb* bacilli in non-necrotic areas, suggesting that increased fumaric acid levels may be seen in patients with higher *Mtb* bacilli counts.

### Significant loss of amino acids, sugars, alcohols, and organic compounds

In TBM cases, the amino acids pipecolic acid and L-pyroglutamic acid were below the GCxGC-TOFMS detection limit. Pipecolic acid is a lysine catabolite and a non-proteinogenic amino acid. It is a naturally occurring amino acid that is commonly elevated in Zellweger syndrome, organic acidemias, and fatty acid oxidation disorders, and is a beneficial metabolite for liver function (Fujita et al., 1999, Vockley, 2021, Zhang et al., 2024). The presence of pipecolic acid is essential for maintaining liver function in severe disease. In our study, pipecolic acid levels were significantly reduced in TBM patients. A study on cerebral malaria suggested that pipecolic acid could be a potential biomarker of disease severity, as it is increased in the plasma of patients with severe cerebral malaria (Beri et al., 2019). L-pyroglutamic acid, also known as oxoproline, is involved in glutathione metabolism and is a marker of glutathione deficiency (Zand Irani et al., 2020). It is also a non-proteinogenic amino acid naturally found in humans (Hundemer & Fenves, 2017). It has been reported as reduced in HIV patients, thereby increasing oxidative stress in humans (Thirion et al., 2024). In animal models, it has been found to block energy production and lipid synthesis in the mouse cerebral cortex (Silva et al., 2001) and is involved in pyroglutamic acidosis, a condition that causes vomiting, nausea, and confusion (Venkataraman et al., 2019). Moreover, L-pyroglutamic acid is formed by the cyclization of glutamic acid, which can occur in FFPE samples.

### Lysine degradation pathway as a possible energy source for *Mtb* persistence

Previous studies have linked the kynurenine pathway to TBM and have shown that quinolinic acid and carnitine can differentiate TBM from other types of meningitis (Isaiah et al., 2024, Isaiah et al., 2024, Plaatjie et al., 2025, Plaatjie et al., 2025). This study of brain tissue does not show that this pathway is significant in *Mtb* pathophysiology; however, it highlights the importance of the lysine degradation pathway (biosynthesized via the aspartate pathway) in TBM. Metabolites linked to this pathway (glutaric acid, pipecolic acid, piperidine, 2-oxoadipic acid, and 2-oxoglutarate) were significantly affected. A previous study emphasized the importance of lysine metabolism to *Mtb* survival, as it is an alternative pathway for energy production through catabolism of lysine, probably through deamination for its carbon and nitrogen source, or the host is compensating for the energy loss by using alternative energy sources, namely lactate produced by glycolysis in the astrocytes to activate microglia cells in a prolonged disease state (Amalia et al., 2022, Mason et al., 2017), which enables the pathogen to persist in a dormant state. It has been hypothesized that to eradicate the disease, inhibition of this pathway could lead to better disease clearance (Hasenoehrl et al., 2019). Figure 8 shows the link between the lysine degradation pathway and the kynurenine pathway. This could mean that the pathway involved in the tissue pathology may differ from the pathway observed in biofluids; however, these pathways can be linked. Also, the type of metabolites observed depends entirely on the equipment’s sensitivity.

### Significant loss of sugars, alcohols, and organic compounds in TBM cases

In our study, glucose levels were approximately 10 mg/g of brain tissue in the non-TBM cases but were below the detection limit in the TBM cases. D-chiro-inositol and myo-inositol are important sugar alcohols involved in glucose regulation in certain diseases, such as diabetes, and exhibit insulin-mimetic properties (Pintaudi et al., 2016, Mancini et al., 2016). In the brain, these metabolites are neuroprotective and have been used to treat depression, and a shortage of these metabolites has been linked to psychiatric diseases (López-Gambero et al., 2020). *Mtb* is said to use these metabolites for its cellular wall composition, development, and virulence (Movahedzadeh et al., 2004, Movahedzadeh et al., 2010). The shortage of these metabolites may indicate their contribution to *Mtb* survival in the TBM host and may also be responsible for the changes observed in mental status, such as confusion and delirium, in patients with severe TBM (Marx & Chan, 2011).

Two alcohols – 2,5-pentadecadien-1-ol and hexanol – were below the detection limit in TBM cases but were present in non-TBM cases (1.79 ± 0.00068 mg/g and 0.68 ± 0.000083 mg/g of brain tissue, respectively). 2,5-Pentadecadien-1-ol, a derivative of 5,10-pentadecadien-1-ol, has antibacterial properties and is used to treat drug-resistant diseases (Nabi et al., 2022, Schmitzer et al., 2022). Hexanol—a derivative of cyclohexane and hexanone—has been identified in breath analyses of pulmonary TB patients as a potential biomarker of TB (Isaiah et al., 2025; Druszczynska et al., 2017). The other alcohols identified as significantly lower in TBM cases were 2-phenylethanol, 6,10,14,trimethyl-2-pentadecanol, undecanol, and tridecan-2-ol. These metabolites are classified as aromatic volatile organic compounds (VOCs) and have antimicrobial properties that have been studied in mycobacterial species to inhibit microbial growth (McNerney et al., 2012, Liu et al., 2014, Kleinwächter et al., 2021, Ndjoubi et al., 2020). However, there are limited studies on TBM regarding these metabolites. 2-Phenylethanol showed strong associations with infarcts in the brainstem and with granulomas in the brain/infratentorial regions, as well as cranial nerve roots. Research on this metabolite, a derivative of phenylethanolamine, is closely linked to epinephrine production, which is critical for neuroprotection. 2-phenylethanol is known to worsen ischemic stroke when in high concentrations; significantly low levels also show this detrimental effect. This could explain the positive correlation with brain infarction when compared in TBM cases (Xu et al., 2019, Hernández et al., 2016, Saavedra & Axelrod, 1973). High levels of 2-phenylethanol may be found in patients with severe brain infarcts, suggesting it may serve as a marker for the presence of bacilli. In the CSF, 2-phenylethanol is metabolized to phenylacetic acid, and in the urine, it is metabolized to hydroxyphenylacetic acid, which is significantly altered in TBM cases (Isaiah et al., 2024, Panoutsopoulos et al., 2004).

### Limitations of the study

There are several limitations to the study, such as the fact that some of the control samples are pathologic and do not represent healthy human brain tissue. Therefore, some of the significant metabolites may be attributable to confounding factors in pathologic non-TBM brain tissue. Other confounding factors that may affect the results are differences in age distribution between the TBM and non-TBM samples. Also, regions from which brain samples were taken differ, some being from the cerebellum, basal stem, spinal cord, midbrain, and cerebral cortex, and this may have effects on the metabolite distribution observed.

FFPE tissue is generally prone to degradation, and the older the FFPE tissue block, the higher the degradation, as the process of fixing involves steps that lead to the loss of some polar metabolites and the binding of functional groups such as carboxamides and amines to endogenous metabolites (Cacciatore et al., 2017). However, larger metabolites such as fatty acids, organic acids, and glycolysis intermediates have previously been identified, and our novel extraction method showed that alcohols, amino acids, and peptides can be extracted from the FFPE tissue; this needs further validation. Identified metabolites will require further validation as they are putatively identified and correspond to MSI level 2.

Analytical variation is unavoidable because the instrument may not adequately detect low-abundance metabolites and because the sample type has low concentrations of the observed analytes. In addition, the number of control samples (*n* = 6) was limited, and the number of blocks (*n* = 36) obtained from this group was used in the statistical analyses rather than the patient numbers.

## Conclusion

Our research, focused on generating hypotheses, suggests a substantial reduction in fatty acids, alcohols, alkanes, alkenes, and metabolites of the lysine degradation pathway, which aligns with previous TBM studies comparing TBM and non-TBM groups in urine and CSF. This shows that although the sample matrices and analytical platforms differ, the results could be linked (though not directly) through the pathway. This suggests that the effect of *Mtb* infection in the host is consistent across all sample types and can be traced. The activity of *Mtb*, although not fully understood, has been shown to significantly decrease metabolite levels, with some metabolites completely depleted in TBM patients. This may be the cause of the symptoms observed in severe TBM cases. The severity of the absence of these metabolites could be an important clue for advanced TBM stages, distinct from other meningeal diseases. Most TBM-based metabolomics studies have been conducted on biological fluids, such as plasma, CSF, and blood; however, to the best of our knowledge, this is the first such analysis of brain tissue. These findings show the importance of using archived tissue materials for further research. In our study, finding healthy FFPE blocks from the 70s to the early 2000s was difficult, as most blocks were archived far from the area where the files were stored and no information was available on their location. We had to include available samples (non-TBM), obtained in the same area as our TBM samples. We also attempted to use (Organization) 1H-NMR (a more specific analytical platform for metabolite identification) as part of the metabolomics analysis. Still, we were unsuccessful due to very limited sample quantity (less than 30 mg), as 1H-NMR is known to lack sufficient sensitivity for small sample quantities. These findings underscore the importance of using archived tissue materials for further research. Despite the availability of these tissue materials, one challenge may be finding completely healthy tissue dated the same year as the older tissue materials. In our study, we used non-TBM samples as controls rather than a completely healthy brain. Further research is needed on TBM brain tissue compared with other disease-related brain tissue to identify features that differentiate TBM from other meningeal diseases and to validate the findings in this paper.

## Supplementary Information

Below is the link to the electronic supplementary material.

Supplementary Material 1

## Data Availability

Will be made available on request from the corresponding author.
