# Supplementary material for: Metabolic insights into the pathophysiology of tuberculous meningitis in FFPE postmortem human brain tissue
Source: Metabolomics. 2026 Aug 7;22(4):138. doi: 10.1007/s11306-026-02511-8 (PMC13451238; doi:10.1007/s11306-026-02511-8)
Supplement: Supplementary file 1 [file 11306_2026_2511_MOESM1_ESM.docx]

**Supplementary Information: Metabolic insights into the pathophysiology of tuberculous meningitis in FFPE postmortem human brain tissue**

Fig. S1. A PCA scores plot showing PC1 (23.3% of the variance) and PC2 (20.1% of the variance), indicating variation between the sample groups: non-TBM controls (red), TBM group (blue), and QC group (green). Principal components 1 and 2 account for 43.4% of the total variation.
